# Does use of pooled cohort risk score overestimate the use of statin?: a retrospective cohort study in a primary care setting

**DOI:** 10.1186/s12875-014-0172-y

**Published:** 2014-11-12

**Authors:** Yook Chin Chia, Hooi Min Lim, Siew Mooi Ching

**Affiliations:** Department of Primary Care Medicine, Faculty of Medicine, University of Malaya Primary Care Research Group (UMPCRG), University of Malaya, 50603 Kuala Lumpur, Malaysia; Curtin Health Innovation Research Institute, Faculty of Health Sciences, Curtin University, GPO Box U1987, 6845 Perth, WA Australia; Department of Family Medicine, Faculty of Medicine and Health Sciences, Universiti Putra Malaysia, 43400 Serdang, Malaysia; Department of Gerontology, Universiti Putra Malaysia, 43400 Serdang, Malaysia

**Keywords:** Pooled cohort, Risk score, AHA/ACC, Statin, Primary care, Atherosclerotic cardiovascular disease risk, ASCVD, CV, Retrospective, Cohort, Malaysia

## Abstract

**Background:**

Initiation of statin therapy as primary prevention particularly in those with mildly elevated cardiovascular disease risk factors is still being debated. The 2013 ACC/AHA blood cholesterol guideline recommends initiation of statin by estimating the 10-year atherosclerotic cardiovascular disease (ASCVD) risk using the new pooled cohort risk score. This paper examines the use of the pooled cohort risk score and compares it to actual use of statins in daily clinical practice in a primary care setting.

**Methods:**

We examined the use of statins in a randomly selected sample of patients in a primary care clinic. The demographic data and cardiovascular risk parameters were captured from patient records in 1998. The pooled cohort risk score was calculated based on the parameters in 1998. The use of statins in 1998 and 2007, a 10-year interval, was recorded.

**Results:**

A total of 847 patients were entered into the analysis. Mean age of the patients was 57.2 ± 8.4 years and 33.1% were male. The use of statins in 1998 was only 10.2% (n = 86) as compared to 67.5% (n = 572) in 2007. For patients with LDL 70-189 mg/dl and estimated 10-year ASCVD risk ≥7.5% (n = 190), 60% (n = 114) of patients were on statin therapy by 2007. There were 124 patients in whom statin therapy was not recommended according to ACC/AHA guideline but were actually receiving statin therapy.

**Conclusions:**

An extra 40% of patients need to be treated with statin if the 2013 ACC/AHA blood cholesterol guideline is used. However the absolute number of patients who needed to be treated based on the ACC/AHA guideline is lower than the number of patients actually receiving it in a daily clinical practice. The pooled cohort risk score does not increase the absolute number of patients who are actually treated with statins. However these findings and the use of the pooled cohort risk score need to be validated further.

## Background

Statins have been extensively studied both in primary and secondary prevention of cardiovascular events [1-4]. Identifying those who need statin therapy is crucial as we need to weigh the cardiovascular (CV) risk against adverse events of drug therapy, so that under- or over-treatment can be minimized. Therefore, cardiovascular risk stratification tools have been developed to help clinicians identify patients, particularly those with mildly elevated cardiovascular risk factors, who should be treated with statins [5].

Until recently, the NCEP ATP-III Framingham risk score was used as a tool to stratify risk for the indication of statin therapy [6]. In November 2013, the American College of Cardiology and American Heart Association (ACC/AHA) released a new guideline for the management of blood cholesterol [7]. Patients with clinical atherosclerotic cardiovascular disease (ASCVD) should receive statin therapy as secondary prevention. For primary prevention, ACC/AHA guideline recommends statin therapy for patients with LDL ≥190 mg/dl. Statin therapy is also recommended for patients with diabetes mellitus and LDL 70-189 mg/dl. Patients without diabetes but with LDL 70-189 mg/dl and a 10-year ASCVD risk ≥7.5% based on the new pooled cohort risk score should be given statin. The pooled cohort risk equation was derived from pooled data of four large cohorts that included both white and black men and women (Framingham and the Framingham Offspring studies, Atherosclerosis Risk in Communities, Cardiovascular Health Study and Coronary Artery Risk Development in Young Adults).

Since the release of the 2013 ACC/AHA guideline there has been a lot of debate and concern about the use of this new pooled cohort risk score as it recommends a much lower threshold of ≥7.5% for the initiation of statins. Hence it is perceived to overestimate CV risk and that more patients will need to be treated with statin [8-10]. Currently, there are still very few studies exploring the use of the pooled cohort risk score in different populations. Hence, we examined the use of the pooled cohort risk score that identifies patients who need statin against patients who are actually receiving statin therapy in a daily clinical practice in primary care.

## Methods

### Setting

This current study is part of a 10-year retrospective cohort study of randomly selected patients registered with a primary care clinic. This clinic is an outpatient clinic of University Malaya Medical Centre, a teaching hospital in Kuala Lumpur, the capital city of Malaysia. This clinic is run by 14 family medicine specialists, 30 vocational trainees in family medicine and other medical officers. This tertiary medical centre including its primary care clinic serves a multi-ethnic population of 450,000 in the surrounding area. The majority of our study the population are middle class patients with around 11 years of formal education. They are also representative of the type of patients that are seen in primary care clinics in Malaysia. Ethics approval was obtained from the Ethics Committee of the University of Malaya Medical Centre.

### Study population

This study is part of a retrospective cohort study for validating the Framingham Risk Score in a primary care Asian population. There were 970 patients in the original cohort and 908 were in the age group 40–75 years (the age group in whom the pooled cohort risk score is applicable according to the ACC/AHA guideline). After excluding patients with incomplete data on LDL level and statin use in 2007, 847 patients were eligible for this analysis.

### Inclusion criteria

Adults aged 40–75 without clinical ASCVD who were already registered in our centre in 1998 were eligible for this study.

### Exclusion criteria

Patients who did not have all the variables to calculate the pooled cohort risk score at baseline were excluded. Those patients with incomplete data on LDL level and statin use in 2007 were excluded as well.

### Data collection

Random numbers were generated by computer based on the patients’ registration number with the clinic. Baseline data was collected in 1998 and follow-up data collected in 2007, a 10-year interval. We extracted the patients’ information from their paper-based records manually. Socio-demographic data and co-morbidities were recorded. Blood pressure was measured by the attending doctor using mercury sphygmomanometer as part of daily routine care. Diagnosis of hypertension in our clinic is made in accordance with standard recommendations i.e. blood pressure ≥140/90 mmHg based on at least 2 blood pressure measurements at least 2 weeks apart [11]. Anti-hypertensive drug use was also recorded.

Diabetes mellitus was defined as documented by the attending physician or the use of hypoglycaemic agents or both. HbA1c levels of diabetic patients were also captured. Smokers were defined if they were still smoking currently. Non-smokers were those who never smoked or currently not smoking regardless of when they had stopped smoking. Renal function was determined by the estimated glomerular filtration rate (eGFR) based on the Cockcroft-Gault formula [12].

Total cholesterol, LDL and HDL cholesterol levels were captured. Statin use in 1998 and 2007 were recorded. We calculated the pooled cohort risk score for those without diabetes but with LDL 70-189 mg/dl using the online pooled cohort risk calculator provided by American Heart Association [13]. ASCVD events from 1998 until 2007 (10-year period) were collected. ASCVD events were defined as nonfatal myocardial infarction (MI), coronary heart disease (CHD) death, nonfatal and fatal stroke in accordance to the ACC/AHA guideline.

### Statistical analysis

All statistical analysis was done using the Statistical Package for Social Sciences (SPSS version 16). Categorical data are reported as proportions (percentage). Mean was used for continuous variables that were normally distributed. Median and interquartile range were used for variables that were not normally distributed.

## Results

847 patients who fulfilled the inclusion criteria were recruited into our analysis. Overall, the baseline mean age of the patients was 57.2 years with 33.1% male. Table 1 shows the cardiovascular risk profile in 1998 and 2007. Only 184 (8%) of patients had chronic kidney disease stage 3 and above in 1998. 86 (10.2%) of patients were on statin therapy in 1998 compared to 572 (67.5%) in 2007.

**Table 1:** **Cardiovascular disease risk profile for adults age of 40 and 75 years in 1998 and their profile in 2007**

**All adults (N = 847)**	**Year 1998**	**Year 2007**
Mean age - yr	57.2 ± 8.4	67.1 ± 8.4
Age <65 (n, %)	672 (79.3)	340 (40.1)
Age ≥65 (n, %)	175 (20.7)	507 (59.9)
Male sex (n, %)	280 (33.1)	280 (33.1)
Ethnicity (n, %)		
Malay	191 (22.6)	191 (22.6)
Chinese	389 (45.9)	389 (45.9)
Indian	256 (30.2)	256 (30.2)
Others	11 (1.3)	11 (1.3)
Mean BMI	26.5 ± 4.64	26.1 ± 4.7
Low-density lipoprotein ≥190 mg/dl (n, %)	153 (11.5)	14 (1.7)
Cholesterol		
Median total – mg/dl (IQR)	232.0 (208.8-259.1)	189.5 (166.3-216.6)
Median low-density lipoprotein – mg/dl (IQR)	158.2 (133.2-181.0)	113.3 (92.0-136.2)
Median high-density lipoprotein – mg/dl (IQR)	46.4 (38.7-54.1)	48.0 (40.8-56.8)
Mean systolic blood pressure - mmHg	140.4 ± 18.3	135.0 ± 16.6
Mean diastolic blood pressure - mmHg	84.65 ± 10.1	78.8 ± 8.2
Patients on antihypertensive agent (n, %)	503 (59.4)	735 (86.6)
Diabetes mellitus (n, %)	379 (44.7)	508 (60.0)
Mean HbA1c - %	7.73 ± 1.8	7.66 ± 1.6
Current smoking (n, %)	61 (6.0)	61 (6.0)
Mean creatinine – umol/L	79.8 ± 23.7	85.2 ± 40.8
Mean eGFR- ml/min per 1.73 m^2^	78.1 ± 26.2 (n = 779)	67.8 ± 27.0 (n = 663)
<30 (n, %)	5 (0.6)	32 (3.8)
30-60 (n, %)	179 (21.1)	254 (30.0)
>60 (n, %)	595 (92.0)	377 (44.5)
Use of statins (n, %)	86 (10.2)	576 (67.5)

IQR Interquartile range.

**Figure 1:**
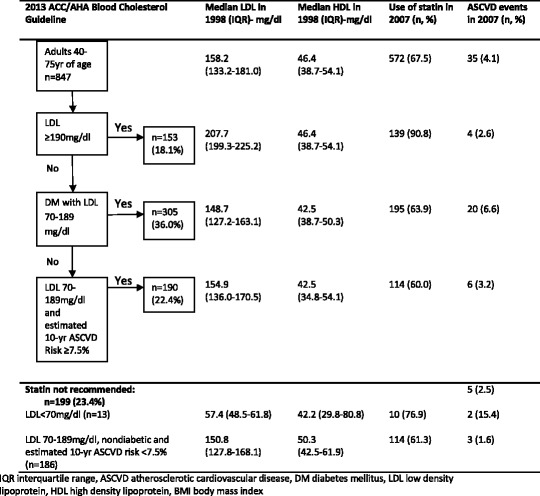
**Comparison of use of the 2013 ACC/AHA blood cholesterol guideline to actual use of statin in a primary care setting and ASCVD events.**

Figure 1 shows the comparison of the ACC/AHA guideline to the use of statins in actual primary care practice. As patients with clinical ASCVD in 1998 were excluded, the first major recommendation group is patients with LDL ≥ 190 mg/dl (n = 153). 90.8% (n = 139) of our patients of this group were on a statin by the end of the 10-year period. For patients with diabetes mellitus and LDL 70-189 mg/dl, only 63.9% (n = 195) of diabetic patients were on statin.

For patients without diabetes but with LDL 70-189 mg/dl, we estimated their 10-year ASCVD risk using the pooled cohort risk score to determine the indication for statin therapy. There were 190 patients with estimated 10-year ASCVD ≥7.5%. Only 60.0% (n = 114) of these patients were receiving statin. Hence, an extra 40% (n = 76) of patients with risk score ≥7.5% need to be treated with statins if the recommendations of the ACC/AHA guidelines were used. On the other hand, 62.3% (n = 124) of patients were receiving statin when it was not indicated based on 10-year ASCVD risk score.

A total of 35 (4.1%) ASCVD events occurred over the 10 year period (31 non-fatal strokes, 1 fatal stroke and 3 non-fatal CHD) (Figure 1). 6.6% of diabetic patients with LDL 70-189 mg/dl had ASCVD events. For those patients with LDL 70-189 mg/dl and estimated 10-year ASCVD risk ≥7.5%, 3.2% had ASCVD events in the 10 years. The pooled cohort risk score overestimated the ASCVD risk in our study population. For those patients in whom statin therapy was not recommended (n = 199), 5 ASCVD events occurred (2.5%).

## Discussion

Clinicians frequently face a dilemma when deciding on statin initiation as primary prevention, particularly in those without diabetes and those with apparently mildly elevated cardiovascular disease risk factors when the indication is less clear and evidence less strong. The recent ACC/AHA guideline suggests patients should be treated with statins if their pooled cohort risk score is ≥7.5%. This lower threshold for initiating statin treatment in contrast to the higher threshold used previously would suggest that many more patients will need to receive statins and that overtreatment will occur. This is of concern particularly as a recent report suggests that subjects with lower risk actually derive no benefit but have more harm from treatment with statins [14]. However the recently updated NICE guidelines also supports initiating statin therapy at lower CV risk i.e. ≥10% albeit using the new QRISK2 score and not the AHA/ACC pooled cohort scoring system. The NICE recommendation has also been supported by others [15,16].

Based on our study, an extra 40% of patients (n = 76) will need to be treated if the pooled cohort risk score was used. This is consistent with a study on use of the 2013 ACC/AHA guideline in the American population based on National Health and Nutrition Examination Survey (NHANES III) where an extra 15.9% of patients, equivalent to 12.8 million more people will need to be treated with statins when compared with the ATP III guidelines [8]. Another study also using the ACC/AHA guideline in a non-American population in Netherlands showed that more adults will also need statin therapy whereby nearly all men and two thirds of women will need to be treated [9].

However in our cohort of patients, we found that more than half of patients (62.3%, n = 124) were actually receiving statin when statin therapy would not have been indicated based on the new pooled cohort risk score recommendations at baseline. However their statin therapy may have been started because they developed diabetes, hypertension or dyslipidaemia which would have increased their CV risk, sometime in the 10 years of follow-up.

Interestingly the number of patients treated “unnecessarily” with statins in actual clinical practice (n = 124) is even higher than the extra number of patients who need to be treated based on recommendations of the new AHA/ACC guideline (n = 76). On balance, the total number of patients recommended by the ACC/AHA guidelines did not exceed the number of patients who were actually receiving statin in real-life daily clinical practice in primary care where most of the lower to moderate risk patients are seen. Perhaps the pooled cohort risk score can guide clinicians in identifying the correct patients for statin therapy, thus avoiding over- or under-treatment. Johansen et al. also pointed out that the pooled cohort risk score may guide the clinicians to focus on patients’ CV risk when initiating statin therapy instead of the lipid profile alone and hence avoid under-treatment in high risk people [17].

According to the NCEP ATP III guidelines, diabetes mellitus is considered a cardiovascular heart disease (CHD) risk equivalent [18]. Clearly this group of patients has high cardiovascular risk and statin therapy will be of benefit to them [19-21]. Our study has also shown that not all patients with diabetes mellitus were receiving statin therapy and this is consistent with findings in other studies [17,22]. Greater effort will be needed to get all patients with diabetes to be on statin therapy.

The pooled cohort risk score is meant to be used to stratify risk for initiation of statin therapy. Although our patients were not risk scored in 1998 for statin use, 60% of them deemed to need statin were eventually receiving some time over the 10 year period. This implies that the use of the risk score identifies patients earlier to receive statins and therefore could be helpful to clinicians in deciding when to start statin.

### Strengths and limitations

A strength of our study is that it is done in a primary care clinic where most patients who are at lower risk for CVD are seen. These are the very same patients in whom initiation of statin therapy as primary prevention is less clear and is when a global CVD risk is most helpful to stratify risk for statin therapy. Furthermore the period studied is relatively long and allows tracking of the use of statin over 10 years. Our study also reflects true clinical practice of the use of statin in primary care. There are several limitations in our study. Firstly, patients with incomplete data, especially HDL cholesterol level which is an important factor in the pooled cohort risk equation, were excluded in this analysis. We also do not have data on when statin was initiated. However patients who needed statins did eventually receive statins some time in the 10-year period.

## Conclusions

An extra 40% of patients need to be treated with statin if 2013 ACC/AHA blood cholesterol guideline is used. However the absolute number of patients needed to be treated based on the ACC/AHA guideline is lower than the actual number of patients who were receiving it in daily clinical practice. The pooled cohort risk score may serve as an appropriate tool to guide clinicians on the initiation of statin therapy. However, further studies are needed to validate the pooled cohort risk equation.

## Abbreviations

ASCVD: Atherosclerosis cardiovascular disease
ACC/AHA: American College of Cardiology and American Heart Association
NCEP ATPIII: National Cholesterol Education Program Adult Treatment Panel III
CV: Cardiovascular
HDL: High density lipoprotein
LDL: Low density lipoprotein
DM: Diabetes mellitus
BP: Blood pressure
